# Teratogenic Effect of Usnic Acid from* Cladonia substellata* Vainio during Organogenesis

**DOI:** 10.1155/2017/5948936

**Published:** 2017-02-27

**Authors:** C. R. Silva, K. S. N. Marinho, T. D. S. Silva, D. K. S. Ferreira, G. M. Aguiar, M. C. B. Martins, K. R. P. Santos, F. C. A. Aguiar Júnior, N. P. S. Santos, E. C. Pereira, N. H. Silva

**Affiliations:** ^1^Laboratory of Chemistry of Natural Products, Department of Biochemistry, UFPE, Recife, PE, Brazil; ^2^Department of Morphology and Animal Physiology, UFRPE, Recife, PE, Brazil; ^3^Laboratory Biotechnology and Pharmaceuticals, Academic Center Academic of Vitória, UFPE, Recife, PE, Brazil; ^4^Department of Geographical Sciences, UFPE, Recife, PE, Brazil

## Abstract

Studies about toxicological potential of usnic acid are limited. This way, the vast majority of data available in the literature are related only to biological activities. This is the first study that aimed to evaluate the oral toxicity of usnic acid during the period of organogenesis. Females rats were distributed in the control groups, treated I and II, at doses of 15 and 25 mg/kg, administered by gavage during the 6° to 15° days of pregnancy. After 20 days the fetuses were removed and analyzed. A reduction in weight gain during pregnancy, increased resorption, reduction in the number of viable fetuses, and their body weight were observed. Morphological changes in the litter were visualized as exposure of the eye and atrophy of the limbs at the dose of 25 mg/kg. Histological analysis of the liver of the fetus showed reduction in the number of megakaryocytes between experimental groups and increase in the number of hepatocytes in a dose of 25 mg/kg. The experimental model used in this study reveals teratogenic effect of usnic acid in the period of organogenesis. Since this achievement, the importance of evaluating the toxic effects of natural substances is imperative, in order to elucidate the care in their indication as drug.

## 1. Introduction

Usnic acid [2,6-diacetyl-7,9-dihydroxy-1,3-dimethyl-8-9b (2H, 9*α*/*β*H)-dibenzo-furandione; C_18_H_16_O_7_] is a compound of natural origin resulting from lichen secondary metabolism. It is considered one of the most important biologically active metabolites with important pharmacological properties: antitumor, antibiotic, antiviral, antioxidant, tuberculostatic, anti-inflammatory, and molluscicide [1–10].

Despite the diverse pharmacological activities attributed to this compound, its use has shown damage to the main organ that acts in the detoxification of endo- and xenobiotics, the liver [11]. It was reported that usnic acid is one of the active ingredients of the supplement Lipokinetix®, causing hepatocellular damage [12]. Data claim that these people who used this product had acute liver failure [13]. Already* in vitro* studies, isolated rat hepatocytes treated with different concentrations of usnic acid showed almost 100% of necrosis [14–16].

The mechanism of action of usnic acid is not fully elucidated. Studies mention this ability of decoupling of the chain of electron transport, affecting mitochondrial function and cellular respiration, effect similar to what happens to carbon tetrachloride [12, 14, 17, 18].

To evaluate the toxicity of naturally occurring substances, such as usnic acid, preclinical trials in order to evaluate the potential of the substance to cause adverse effects on exposed organisms are included, in particular the effects of exposure during pregnancy which is one of the basic requirements for the use of bioactive molecules [19, 20].

Experimental studies using animal models, traditionally, provide the basis for screening teratogenic potential of substances to the verification of a particular agent to be considered toxic. These investigations have the key role in the elucidation of the principles and mechanisms involved in teratogenesis, which may be related to drug exposure conditions, stage of development of the embryos, and pathogenic mechanism of each agent [21].

**Figure 1:**
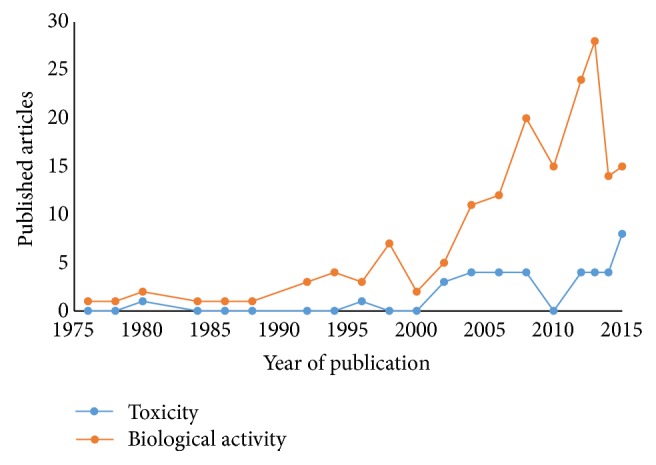
Scientific research on biological activity and toxicity of usnic acid. Source: Scopus.

In recent years there has been a breakthrough in the number of scientific researches to demonstrate the effectiveness of the various biological activities attributed to usnic acid (Figure 1).

However, studies about its toxicological potential are limited, so that the vast majority of data available in the literature are related only to the biological activities of compound [22–26].

Therefore, this is the first study that aimed to evaluate the oral toxicity of purified usnic acid from* Cladonia substellata* on the reproductive development of female rats during the period of organogenesis.

## 2. Material and Methods

### 2.1. Lichens Samples

Samples of* Cladonia substellata* Vainio were collected on sandy soils of “tabuleiro,” savannah like vegetation, in Atlantic rainforest domain, along of Federal highway (BR 101), in the municipality of Mamanguape, Paraíba State, Northeast Brazil, coordinates 06°42′42,4′′S and 35°07′07,0′′WGr.

The usnic acid, the main substance of the species, was extracted using the eluotropic series of solvents, diethyl ether, chloroform, and acetone [27]. Sequentially, it was isolated and purified in silica gel column from ether extract and characterized as preestablished methodology in the Natural Products Laboratory of the Department of Biochemistry, through analyses of HPLC, ^1^HNMR, and melting point [10]. Quantitation of usnic acid was carried out through HPLC, by injecting different amounts of purified usnic acid, and calculated by direct calibration. In addition, optical rotation of usnic acid was determined in a Jasco P2000 polarimeter in Analytical Centre of Fundamental Chemistry Department of Federal University of Pernambuco.

### 2.2. Experimental Animals

Wistar rats (*n* = 18) provided by the biotherium of the Department of Nutrition at the Federal University of Pernambuco, weighing about 300 g with 60 days of life, were used. The animals were kept in cages lined with air-conditioned environment in photoperiod (12 h light/12 h dark) at a controlled temperature of 25 ± 2°C with exhaust air and free food access ad libitum water. The experimental protocols were approved by the Ethics Committee of the Federal University of Pernambuco (process number 23076.029828/2013-94).

### 2.3. Experimental Procedure

Initially, females were submitted to the study of the estrous cycle in order to determine the fertile period. The analysis was carried out by vaginal swab technique for obtaining slides containing vaginal epithelial cells which were stained by hematoxylin-Shorr. Subsequently, using an Olympus microscope (BH-2, Japan) was identified the period of ovulation of the female, called estrus in which the females were matched with male and the mating was confirmed by the presence of “plug” (whitish mass spermatozoa in the vaginal opening) or the presence of sperm in the vaginal smear. The first 24 h after confirmation of mating were considered day zero (D0) of gestation. Females were randomly distributed in the control groups (*n* = 6) treated I (*n* = 6) and treated II (*n* = 6). The control group received 1 mL of physiologic solution, while the treated groups I and II received the purified usnic acid in doses of 15 mg/kg and 25 mg/kg body weight, respectively. Doses were based on 50% of LD_50_ (lethal dose that kills 50% of the animals) determined to rats [28], and 25 mg/kg, as recommended by Merck® index [29]. The doses of purified usnic acid were dissolved in 10 mL of PBS buffer Merck, and the product was administered orally (gavage), in daily doses, from 6th up to 15th day of pregnancy. During administration females were weighed to assess body mass gain at 0°, 6°, 10°, 14°, and 20 days of gestation. After this period, the mice were euthanized by overdose of the anesthesia with Urethane (1.25 mL/kg). Uteri were dissected, and after opening them, fetuses were removed and examined in the craniocaudal direction, to evaluate the presence of external malformations. Then, fetuses and their placentas were weighed.

The deployment sites were identified by iodine solution (Lugol 2%). In free wombs of their content the deployment sites were visualized by contrast to the uterine tissue. Deployments appeared as bright spots that were located and accounted for. The number of resorptions was the result of the number of implantations sites minus the number of fetuses.

### 2.4. Histomorphometric Analysis

For hepatotoxicity registration after euthanasia the livers of pregnant rats and fetuses were dissected and weighed and then subjected to histopathological analysis. The collected material was kept in buffered formalin solution (10%) for 24 h and then processed for routine histological technique. The blocks were cut into 4 mm thick, by Manson trichrome stained, and analyzed by light microscopy (Olympus BH-2, Japan).

For histomorphometric analysis slides were photographed under fixed focus and clarity of field through Motic® Images Plus 2.0 software with a digital camera attached to an optical microscope (Olympus BH-2, Japan) and connected to the computer. We obtained 30 photomicrographs per slide at full 400x magnification.

The photomicrographs were submitted to appropriate measurements using ImageJ software version 1:44 (Research Services Branch, U.S. National Institutes of Health, Bethesda, MD, USA). For the fetus, the following measurements were considered: number of hepatocytes (NH) and number of megakaryocytes (NM), and for pregnant rats: number of hepatocytes (NH) and number of Kupffer cells (NCK).

### 2.5. Statistical Analysis

Data were expressed as mean ± standard deviation (SD). Statistical significance was determined by one-way ANOVA followed by Tukey's test *P* < 0.05 significant analysis. All analyzes were performed using Prism software (GraphPad Software, Inc., San Diego, CA, version 5.01).

## 3. Results

### 3.1. Chemical Analyses of Usnic Acid

Data of extraction, purification, and spectroscopic analyses of usnic acid used in this study were previously described [10]. The optical rotation was *α*_25_^*D*^: +478.2200 (c 1.0 acetone). This way, the usnic acid used in our study is dextrorotary.

**Figure 2:**
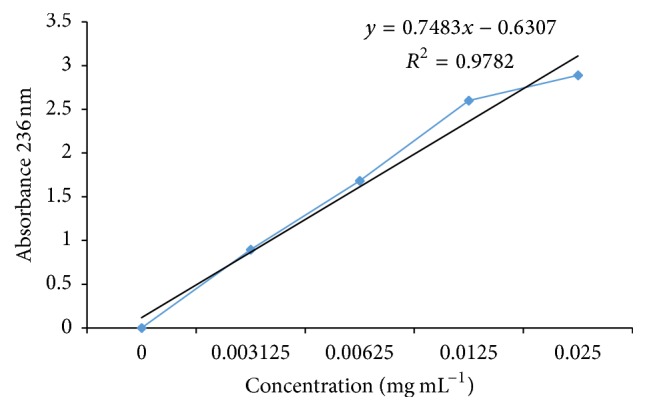
Calibration line of purified usnic acid from* Cladonia substellata*.

The thallus of* C. substellata *presented 95% of usnic acid, by direct calibration (Figure 2), and HPLC analyses showed 95% of purity.

### 3.2. Reproductive Variables Attributed to Pregnant Rats

**Figure 3:**
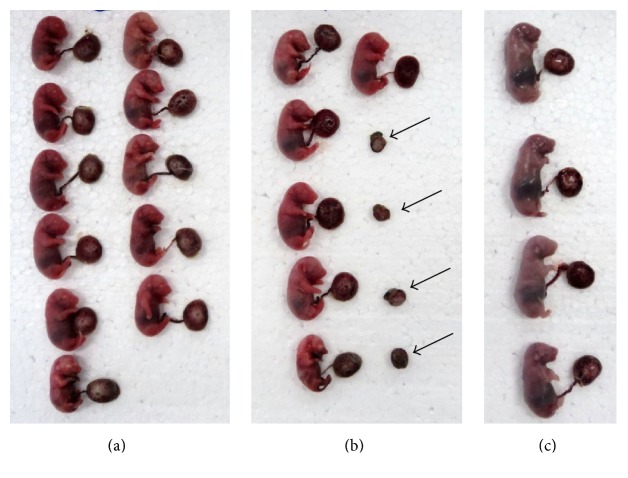
Fetuses obtained from experimental and control groups and treated at 25 mg/kg: (a) Control group, (b) treated group at 25 mg/kg. Arrows showing embryos with interruption in the development and tiny placentas, (c) treated at 25 mg/kg presenting reduction in the number of viable fetuses.

During oral administration of purified usnic acid at 15 mg/kg and 25 mg/kg, no death was registered during treatment. The pregnant rats in the experimental groups showed a significant reduction in body weight gain at 10, 14, and 20 days of gestation (Table 1). With respect to the total number of fetal resorptions, an increase in the group exposed to the dose of 25 mg/kg, the presence small placentas, and further reduction in the number of fetuses were observed (Figure 3). The group exposed at 15 mg/kg remained close to the control group (Table 1).

### 3.3. Assigned Parameters to Prole

The treatment with usnic acid changed the parameters assigned to the offspring (average body weight, average weight of the livers, and external malformations). The average weight of fetuses and their placenta showed decrease by approximately 20% at 15 mg/kg, while at 25 mg/kg a reduction of about 39% was observed (Table 2). At 15 mg/kg a 32% reduction in the weight of fetal livers was observed. By the other hand, a relevant reduction of about 60% was observed in animals treated at 25 mg/kg (Table 2).

**Figure 4:**
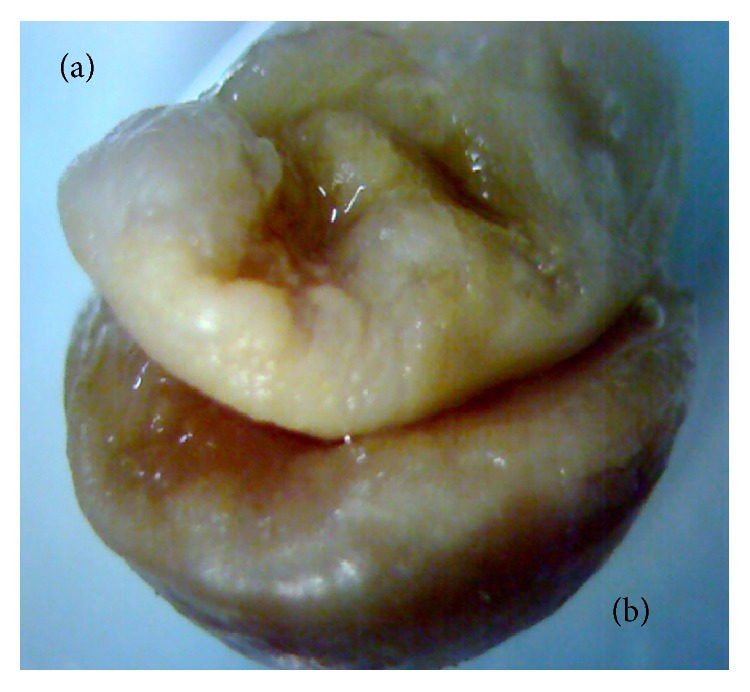
Fetus obtained from female treated with purified usnic acid at 25 mg/kg presenting interruption in embryonic development: (a) mass developing and (b) placenta.

**Figure 5:**
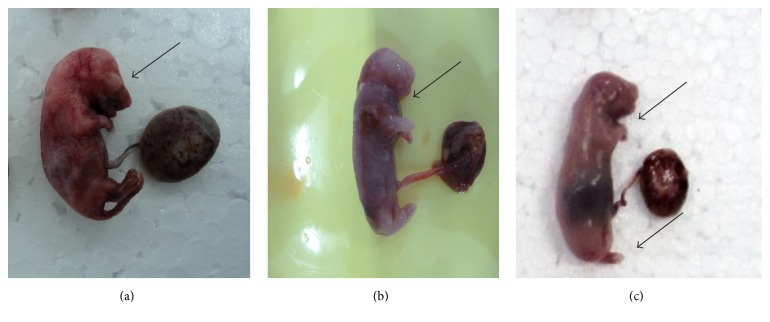
Malformations in fetuses obtained from treated females with purified usnic acid at 25 mg/kg: (a) Arrow showing protrusion of the eyeball, (b) Arrow showing cell mass in the upper region of the fetus, (c) Arrows showing atrophy of the upper and lower limbs.

Concerning the external morphology, the fetuses treated at 25 mg/kg presented with abnormal patterns with disruption in their development (Figure 4), protrusion of the eyeball (Figure 5(a)), substantial mass proliferation in the region top of the face and neck (Figure 5(b)), and atrophy of the upper and lower limbs (Figure 5(c)). At 15 mg/kg none of those changes were displayed.

In the microanatomic examination of the liver of experimental groups, liver lobes consisting of cords of hepatic cells anastomosed to capillary sinusoids which were covered by endothelial cells and Kupffer cells were exhibited. A limit clear between the hepatic lobules with typical organization structures of port spaces was observed. It was not possible to visualize the space of Disse and Ito cells, indicating that there was no damage to the liver structure.

The liver of fetuses from experimental groups showed anastomosing cords of hepatic cells to capillary sinusoids. However, it was not possible to identify a lobular organization, which is considered normal for this gestational age (20 days). The sinusoid capillaries were completely filled with blood, preventing the display of Ito cells and Kupffer cells. The structures of door spaces were not identified. Among the liver cells a large number of blood cell lineages were found at different stages of maturation. Among these, the megakaryocytes were easily identifiable.

### 3.4. Histomorphometric Analysis

The results from the histomorphometric analysis of the liver of pregnant rats exposed to usnic acid demonstrated significant changes in the average of the total number of hepatocytes per animal. The animals exposed at 15 mg/kg showed an increase of about 21%, while those exposed at 25 mg/kg had a 29% increase (Table 3).

The count of Kupffer cells for animals exposed at 15 mg/kg did not show any change in the total number of such cells per animal. The animals treated at 25 mg/kg showed an increase of about 53%, when compared to the control group (Table 3).

The histomorphometry of fetus liver relative to the total number of hepatocytes did not show any change in the population of these cells at 15 mg/kg. In contrast, at 25 mg/kg a mean increase of approximately 24% was achieved. Regarding the counting of megakaryocyte, doses of 15 mg/kg and 25 mg/kg caused a decrease of about 30% and 53%, respectively (Table 4).

## 4. Discussion

The studies related to the toxicology of development using natural products are concentrated in the predeployment phase,* in vitro* culture of embryos in animal models, and in physical maturation and postnatal of the descendants. Little is elucidated with respect to the study of toxic action during the period of organogenesis, which is considered the most important of the intrauterine stage [30–34].

The assessment of the effects of a drug on the reproductive period includes research to analyze toxicity on the maternal organism and its offspring. Signs of maternal toxicity may be related to the reduction of body weight, followed or not by a decrease in food consumption; changes in weight and/or morphology of the bodies; and the occurrence of deaths during the treatment period. Our results showed that administration of usnic acid in both doses tested interfered in maternal weight gain during pregnancy, signaling an indication of maternal toxicity of the compound [35].

Similar results can be found in reproductive toxicity studies with *β*-lapachone, natural compound which has some pharmacological properties similar to usnic acid. Exposure to *β*-lapachone at doses of 40 mg/kg, 60 mg/kg, and 160 mg/kg for organogenesis is able to reduce body weight gain during pregnancy, induce the onset of skeletal defects in offspring, and decrease the number of fetuses per pregnancy [36].

Reduction in the number of viable fetuses per pregnancy, that is, those who continued the development after birth, is outside the normal range, because according to Krinke [37], Wistar rats of childbearing age have an average of 12 to 14 fetuses per litter.

Many of the changes observed in fetuses during intrauterine development can arise as a result of maternal exposure to teratogenic agents. This way, there is no necessarily a direct effect on progenitors. The effects of chemicals or compounds of natural origin during pregnancy can manifest as miscarriages, abnormalities, and delayed development. A very significant reduction in body weight of the conceptus is a fetotoxicity indication, inducing a delay of intrauterine development, since according to Sharp and Laregina [38], fetal body weight at birth is around 6 g.

Interruptions in intrauterine development, when usnic acid was administered at 25 mg/kg, were similar to reproductive toxicity studies of* Duguetia furfuracea* extracts. This product (1 mL), when administered orally during the preimplantation period to complete organogenesis, caused a direct effect on fetuses, delayed fetal development, presence of placenta atrophied without fetus, which clearly indicates that this was reabsorbed, and beyond external malformations, which are most obvious changes during the period of organogenesis [39].

The decrease in the weight liver of fetuses is an indication of hepatotoxic action of the usnic acid, in view of the natural and/or synthetic substances that can easily penetrate the fetal circulation through the liver before reaching the systemic circulation, and this exposure can trigger hepatotoxic effects on individuals exposed [40].

In microanatomic analyses of the liver of all animals treated with usnic acid no change was observed in hepatic tissue, such as necrotic areas. On the other hand, some studies have shown extensive lesions in the liver of animals subjected to subchronic exposure of usnic acid [4].

Histomorphometric analysis reveals a hepatotoxic effect induced by the administration of usnic acid at 25 mg/kg, triggering cell proliferation of hepatocytes for a possible regeneration of liver parenchyma, a regenerative mechanism involving the increase in the number of these cells. The abnormal proliferation of liver cells can occur in various pathological and/or experimental situations, influenced by many factors, and in the liver an immense regenerative capacity when submitted to aggression. The regenerations of the hepatic tissue are dependent on 70% of proliferation of hepatocytes to restore normal liver architecture, in pathological conditions [41–43].

The increase in Kupffer cells in the hepatic tissue may be involved in the pathogenesis of some liver injury. According to some authors, these cells release biologically active substances, such as cytokines, which promote the pathogenic process in the tissue immediately after activation [44–46]. The morphometric study of Kupffer cells to pregnant rats exposed at 25 mg/kg is an indication of inflammation and increased liver damage due to exposure to the compound. The megakaryocytes are responsible for originating the blood platelets [43]; the reduction of multinucleated cells, megakaryocytes, in the offspring of the experimental groups confirms the toxicity of usnic acid, by changing the process of formation of these cells which can lead to a deficiency in the early stages that takes the route of blood coagulation in the body.

## 5. Conclusion

The experimental model used in this study reveals teratogenic effect of usnic acid in the period of organogenesis, and the dose of 25 mg/kg was considered the more toxic, being the dose of 15 mg/kg a candidate for new experimental research. It reveals the importance of the evaluation of the toxic effects of natural substances in order to elucidate the care in their indication as drug, particularly during pregnancy.

## Figures and Tables

**Table 1:** Reproductive variables attributed to pregnant rats.

	Groups		
	G1 (Control)	G2 (15 mg/kg)	G3 (25 mg/kg)
Mean body weight (g)			
Day 0	208 ± 4.3	209 ± 5.6	208 ± 5.2
Day 6	227 ± 1.4	227 ± 3.1	226 ± 5.8
Day 10	255 ± 3.4	236 ± 3.8^*∗*^	224 ± 5.8^*∗∗∗*^
Day 14	276 ± 2.3	250 ± 2.8^*∗∗∗*^	229 ± 6.9^*∗∗∗*^
Day 20	299 ± 2.5	279 ± 3.6^*∗∗∗*^	247 ± 6.6^*∗∗∗*^
Number of implantations	12 ± 0.5	10 ± 1.0^*∗*^	8 ± 1.4^*∗∗∗*^
Number of resorptions	1.1 ± 0.4	3.5 ± 0.5^*∗*^	5.0 ± 1.0^*∗∗∗*^

Data are expressed as mean ± standard deviation. ANOVA-Tukey. ^*∗*^*P* < 0.05, ^*∗∗∗*^*P* < 0.001 compared to the control group.

**Table 2:** Parameters of prole.

	Groups		
	G1 (Control)	G2 (15 mg/kg)	G3 (25 mg/kg)
Mean body weight (g)	5.7 ± 0.3	4.6 ± 0.1^*∗*^	3.5 ± 0.1^*∗∗∗*^
Liver weight	0.5 ± 0.02	0.3 ± 0.02^*∗*^	0.2 ± 0.05^*∗∗∗*^

Data are expressed as mean ± standard deviation. ANOVA-Tukey. ^*∗*^*P* < 0.05, ^*∗∗∗*^*P* < 0.001 compared to the control group.

**Table 3:** Histomorphometric analysis of the liver of pregnant rats treated with usnic acid.

	Groups		
	G1 (control)	G2 (15 mg/kg)	G3 (25 mg/kg)
NH	90.44 ± 15,61	109.38 ± 16,02^*∗*^	116.96 ± 12.67^*∗∗∗*^
NCK	3.26 ± 2.26	4.03 ± 2.62	5 ± 2.83^*∗∗∗*^

NH: number of hepatocytes; NCK: number of Kupffer cells. Data are expressed as mean ± standard deviation. ANOVA-Tukey. ^*∗*^*P* < 0.05, ^*∗∗∗*^*P* < 0.001 compared to the control group.

**Table 4:** Histomorphometric analysis of the liver of the fetus from rats treated with usnic acid during pregnancy.

	Groups		
	G1 (control)	G2 (15 mg/kg)	G3 (25 mg/kg)
NH	33.4 ± 8.6	33.2 ± 9.9	41.6 ± 6.0^*∗∗∗*^
NM	0.17 ± 0.01	0.12 ± 0.01^*∗*^	0.08 ± 0.01^*∗∗∗*^

NH: number of hepatocytes; NM: number of megakaryocytes. Data are expressed as mean ± standard deviation. ANOVA-Tukey. ^*∗*^*P* < 0.05, ^*∗∗∗*^*P* < 0.001 compared to the control group.
